# Raffinose in *Brassica* Crops: Integrating Plant Stress Physiology, Food Quality, Nutrition and Precision Breeding

**DOI:** 10.3390/ijms27219659

**Published:** 2026-10-28

**Authors:** Maria Czernicka, Małgorzata Czernicka

**Affiliations:** 1 Department of Bioenergetics, Food Analysis and Microbiology, Faculty of Technology and Life Sciences, University of Rzeszów, Zelwerowicza 8b St., 35-601 Rzeszow, Poland; 2 Department of Plant Biology and Biotechnology, Faculty of Biotechnology and Horticulture, University of Agriculture in Krakow, al. Mickiewicza 21, 31-120 Krakow, Poland

**Keywords:** *Brassica*, CRISPR/Cas, galactinol synthase, marker-assisted selection, NGT, polyploidy, raffinose-family oligosaccharides, stress tolerance, food quality

## Abstract

Raffinose-family oligosaccharides (RFOs) have nutritional and physiological significance that cannot be inferred from concentration alone. In *Brassica*, interpretation of their food value and definition of breeding targets require consideration of edible organ, environmental history, analytical resolution, and intended use. This critical review integrates evidence on RFO composition, plant physiology, food quality, and nutrition with an evidence-weighted assessment of conventional breeding, genetic transformation, and new genomic techniques. Direct *Brassica* experiments are distinguished from evidence derived from soybean and *Arabidopsis* and from prospective applications. Direct studies demonstrate that RFO metabolism in *Brassica* is manipulable, but they do not support a universal strategy for improving vegetable or meal quality. Genome-editing studies in soybean further show that reductions in total RFOs may coexist with increased raffinose and that lower RFO concentrations do not themselves demonstrate nutritional benefit. Tissue-specific regulation may offer a route to balance seed composition with stress responses, although genotype-dependent regeneration remains an important constraint. A three-tier framework is therefore proposed in which raffinose is interpreted as a stress-responsive metabolite, a potential food-quality indicator, and a context-dependent nutritional component. Compound-specific phenotyping, functional validation, and product-relevant physiological testing should precede deployment of low-RFO or stress-responsive *Brassica* material.

## 1. Introduction: Why Raffinose Requires a Context-Dependent Interpretation

Raffinose and related RFOs are frequently discussed as anti-nutritional factors in food and feed research, while their physiological functions in plants and the context dependence of their nutritional effects require a more differentiated interpretation [1,2,3]. Conclusions derived from legume seeds should not be transferred directly to edible *Brassica* organs without accounting for analyte identity, exposure and food matrix. In particular, grouped raffinose–stachyose and broader oligosaccharide measurements cannot establish the contribution of individual raffinose to dietary exposure [4,5].

Raffinose (α-D-galactopyranosyl-(1→6)-α-D-glucopyranosyl-β-D-fructofuranoside) is the prototypical member of the RFO series, a homologous family of galactosylated sucrose derivatives that includes stachyose, verbascose, and higher-degree-of-polymerization RFOs [3,6]. The biosynthetic sequence begins with the conversion of myo-inositol and UDP-galactose to galactinol by galactinol synthase (GolS), followed by galactose transfer from galactinol to sucrose by raffinose synthase (RFS) [7,8]. The pathway is ancient, broadly conserved in the plant kingdom, and encoded by multigene families subject to tissue- and stress-specific regulation [9,10]. In *Arabidopsis thaliana*, the *AtGolS* gene family exhibits stress- and tissue-dependent expression patterns in response to cold, drought, heat, and oxidative stress, establishing galactinol and raffinose as central nodes in abiotic-stress metabolism [10,11]. The biochemical versatility of this pathway renders its products simultaneously relevant to stress tolerance, carbon storage, and carbon allocation during seed maturation [3,6].

The genus *Brassica* (family Brassicaceae) encompasses a wide diversity of edible organs: leaves (kale, cabbage, Brussels sprouts), inflorescences (broccoli, cauliflower), stems (kohlrabi, kale stems), roots (turnip, swede), and oilseeds (canola/rapeseed). Each organ presents a distinct physiological and metabolic context for raffinose accumulation [12]. Edible-leaf *Brassica* crops are exposed to diurnal and seasonal temperature fluctuations that can promote RFO accumulation in vegetative tissues [13,14], while oilseeds accumulate RFOs during late seed maturation, with BnHSFA4a-mediated regulation of *BnGolS1* contributing to this process [15,16]. These distinct tissue contexts motivate organ-specific interpretation rather than a single crop-wide RFO target [2].

Three converging developments motivate the present re-evaluation. First, the genome-wide identification of 13 annotated *BnaRFS* family members in *B. napus* [17] and the functional characterisation of BnHSFA4a [16] have provided a scaffold for interpreting *Brassica*-specific RFO regulation. Second, the International Scientific Association for Probiotics and Prebiotics (ISAPP) consensus definition of dietary prebiotics [18], together with expanding evidence for microbiota-dependent utilisation of fermentable carbohydrates [19,20], requires a more critical assessment of the nutritional interpretation of *Brassica* raffinose. Third, activity-based metaproteomic work in the mouse gut microbiome has characterised candidate microbial α-galactosidases relevant to RFO catabolism [21], providing a basis for understanding inter-individual variability in response. Against this background, the continued application of a legume-derived anti-nutritional framework to *Brassica* vegetables appears increasingly inconsistent with the available physiological, analytical, and nutritional evidence.

Accordingly, this review integrates physiological, compositional, analytical, nutritional, and breeding perspectives to reassess the significance of raffinose in *Brassica* vegetables. Particular emphasis is placed on distinguishing direct evidence from edible *Brassica* tissues from findings derived from *B. napus*, model plants, other crops, purified RFO substrates, and microbiome studies. The analysis further considers how genotype, tissue identity, environmental exposure, postharvest handling, processing, analytical methodology, dose, and host context jointly determine the biological and nutritional interpretation of raffinose. The aim of this critical review is therefore to determine whether the conventional classification of raffinose as an anti-nutritional constituent is supported by the available evidence for *Brassica* vegetables and to propose a context-dependent framework for its interpretation across plant physiology, food quality, and nutrition.

Unlike earlier reviews centred on general RFO biology, plant stress responses, or legume-dominated nutritional evidence [2,3,22,23], this critical synthesis uses edible *Brassica* organs as its primary application context. Its particular contribution is to connect (i) the strength and biological proximity of molecular and compositional evidence, (ii) the distinction between compound-specific raffinose and grouped oligosaccharide fractions, (iii) preharvest cold acclimation versus postharvest refrigeration, and (iv) the nutritional and organ-specific breeding implications of those distinctions. This framework is interpretative, not a claim that the underlying biosynthetic pathway or the broader RFO stress–nutrition trade-off is newly discovered. Available direct compositional evidence remains concentrated in kale, whereas molecular interventions are dominated by oilseed rape, and these imbalances limit generalisation to other edible *Brassica* organs.

## 2. Literature Selection and Evidence Weighting

This review was conducted as a targeted critical synthesis, not a systematic review or meta-analysis. The relevant literature was identified through topic-focused searches of Scopus, Web of Science, PubMed and ScienceDirect, covering publications available up to July 2026. Search combinations included “raffinose”, “raffinose family oligosaccharides” and “RFOs”, together with “*Brassica*”, “*Brassica oleracea*”, “*Brassica napus*”, “kale”, “cabbage”, “broccoli” and thematic terms such as “cold acclimation”, “cold storage”, “postharvest”, “FODMAP” (fermentable oligosaccharides, disaccharides, monosaccharides and polyols), “prebiotic”, “microbiome”, “α-galactosidase”, “HPAEC-PAD” (high-performance anion-exchange chromatography with pulsed amperometric detection), “GC-MS” (gas chromatography–mass spectrometry) and “HILIC-MS” (hydrophilic interaction liquid chromatography–mass spectrometry). Reference lists of relevant primary studies and reviews were also examined. Original articles and, where appropriate, publisher or institutional records were consulted to check study identity, experimental context and reported outcomes. The aim was a purposeful, critically appraised selection, not exhaustive coverage of the literature.

Inclusion was guided by relevance to raffinose or related RFOs, biological proximity to *Brassica* crops and edible organs, and the availability of identifiable analytes, methods or experimental outcomes. Primary studies directly examining edible *Brassica* tissues were prioritised for food-related conclusions. Studies of *Brassica napus* seeds and other *Brassica* tissues provided crop-specific context; evidence from *Arabidopsis*, soybean, other legumes and model systems was used for mechanistic interpretation or breeding proof-of-concept without assuming transferability to edible *Brassica* vegetables. Evidence on isolated RFOs, microbial fermentation, animal models and human dietary interventions was interpreted according to its experimental context rather than equated with effects of consuming characterised *Brassica* foods. Secondary reviews supplied background but were not counted as independent experimental evidence; reports lacking traceable or relevant primary findings were not used to support specific quantitative or causal claims. Studies reporting combined raffinose–stachyose, total RFO or broader oligosaccharide fractions were retained where informative, particularly for assessing reporting limitations, but their results were not interpreted as individual raffinose concentrations.

Evidence was assessed qualitatively along two distinct dimensions: biological relevance (species, organ, experimental system and intended application) and methodological strength (analyte specificity, appropriate replication and controls, analytical validation, limits of detection (LOD) and quantification (LOQ), recovery, reporting on a fresh- or dry-weight basis, uncertainty and functional confirmation where applicable). These considerations informed the strength and scope of interpretations rather than functioning as mandatory exclusion criteria or a numerical quality score. Relative metabolite abundance was distinguished from absolute compound concentrations, associations from experimentally demonstrated functions, and direct *Brassica* breeding interventions from cross-species proof-of-concept and proposed applications. Where crop- or tissue-specific evidence was insufficient, this limitation was stated rather than resolved by extrapolation. For the intervention comparison, compositional changes, measured physiological or nutritional outcomes and proposed applications are reported separately. A changed sugar profile is not itself a cultivar-level breeding outcome [15,17,24,25,26,27,28].

## 3. The Physiological Logic of Raffinose Accumulation

Galactinol synthase (GolS; UDP-galactose:myo-inositol galactosyltransferase, EC 2.4.1.123) catalyses the formation of galactinol and UDP from UDP-galactose and myo-inositol [3,8,9]. Raffinose synthase (RFS; EC 2.4.1.82) transfers a galactosyl group from galactinol to sucrose, yielding raffinose and myo-inositol; stachyose synthase (STS; EC 2.4.1.67) similarly extends raffinose to stachyose, while further elongation may yield verbascose through species-dependent enzyme activities [3,29]. Myo-inositol released in these reactions can be reused by GolS and is not directly recycled into UDP-galactose. Galactinol is a galactosyl donor, not a demonstrated rate-limiting intermediate. Evidence linking RFO metabolism to abiotic stress derives mainly from *Arabidopsis* and other model plants, where GolS-associated responses vary with stress and tissue [7,10,30]. These mechanisms cannot be assumed to operate identically in edible *Brassica* organs [2,6,29,31]. Gene and transcript symbols are italicised; protein and enzyme designations are in roman type. RFS and RS are retained as published aliases for raffinose synthase; sequence accessions and genome locus identifiers are distinguished from gene symbols.

Lang et al. [16] established a specific regulatory connection involving *Brassica napus*: BnHSFA4a binds heat-shock elements in the *BnGolS1* promoter, as demonstrated by yeast one-hybrid assays, while promoter-reporter experiments confirmed promoter activity. *BnHSFA4a* expression during seed development preceded increases in GolS activity and galactinol accumulation. Functional tests of desiccation tolerance, including overexpression and complementation, were also performed in *Arabidopsis*. Thus, the evidence supports BnHSFA4a–*BnGolS1* promoter binding in a defined experimental context, not direct regulation of RFS or STS throughout *Brassica* or a demonstrated benefit of increased raffinose in edible leaves and inflorescences [16]. This specific connection is described as BnHSFA4a-mediated regulation of *BnGolS1* during seed desiccation responses.

**Figure 1:**
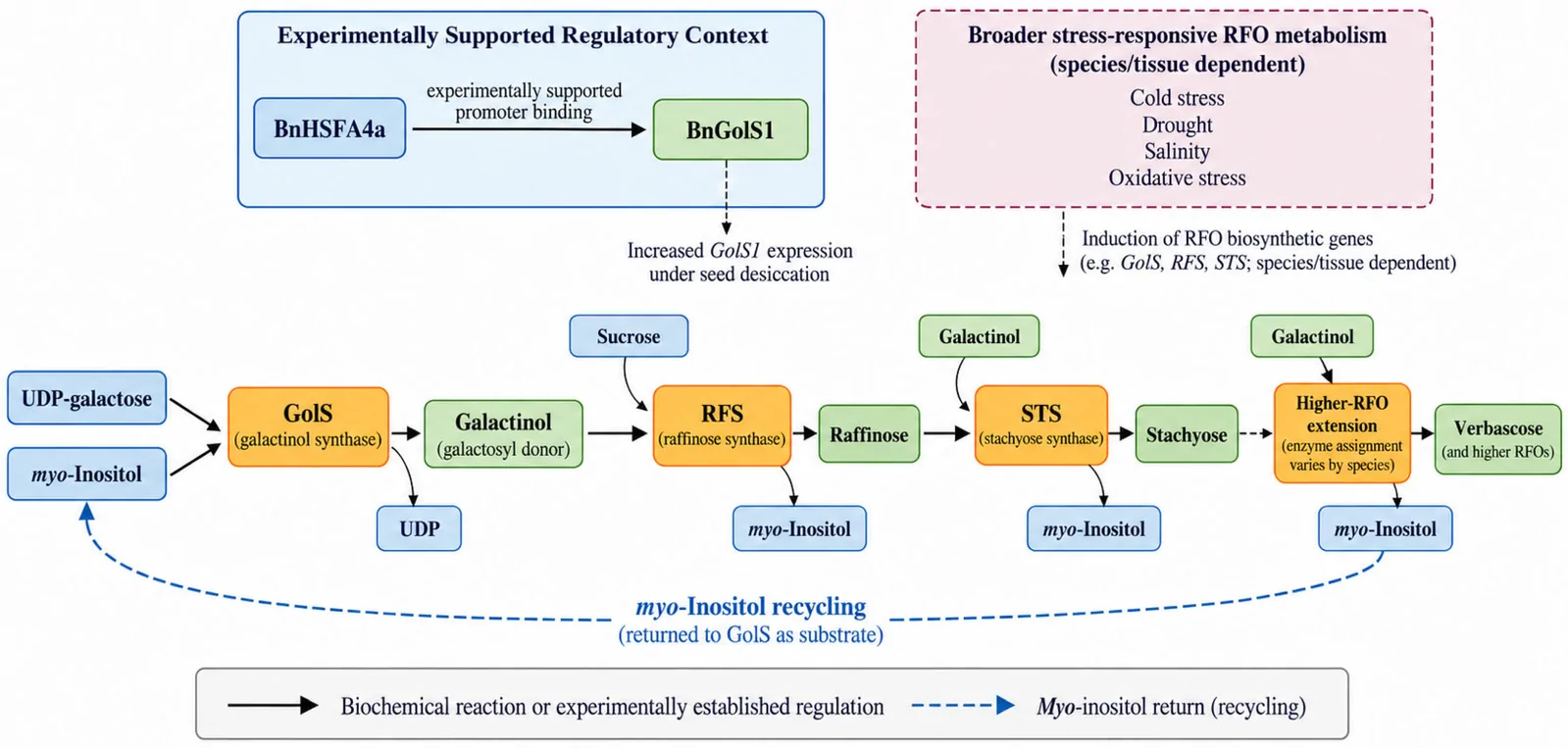
Core reactions of galactinol-dependent RFO biosynthesis and evidence-bounded regulatory context. GolS produces galactinol from UDP-galactose and myo-inositol, and galactinol subsequently acts as the galactosyl donor for raffinose, stachyose, and higher RFO synthesis, with myo-inositol released and recycled. The BnHSFA4a–*BnGolS1* interaction represents experimentally supported promoter binding in *Brassica napus* associated with seed desiccation, whereas the broader stress-responsive module illustrates conceptual, species- and tissue-dependent regulation rather than a validated direct regulatory cascade. Sources: Sengupta et al. [3], Lang et al. [16], and Liu et al. [22].

The allopolyploid genome of *B. napus* provides a complex context for metabolic gene families [29,30]. Zhou et al. [17] identified 13 *BnaRFS* family members, with six entries each for *B. rapa* and *B. oleracea* in Table 1 and Results (the abstract instead reports four for *B. rapa*) and reported that *BnaRFS6* overexpression in *B. napus* altered stalk carbohydrate composition. The reported mitochondrial localisation was tested in *Arabidopsis* protoplasts rather than established by direct imaging of every *Brassica* organ. Family expansion does not demonstrate equal activity, redundancy or physiological function for all members; the breeding implications are considered in Section 7.2. Figure 1 summarises the core reactions and the narrower regulatory connection supported by the studies reviewed here.

Evidence from edible *Brassica* tissues is substantially more limited but provides an important bridge between molecular mechanisms and food composition. Santarius and Milde [31] showed that frost hardening in cabbage (*B. oleracea*) was associated with changes in soluble-carbohydrate distribution, including a physiologically relevant raffinose pool in the chloroplast fraction. This remains one of the clearest direct *Brassica*-specific observations linking raffinose localisation with cold adaptation. Studies in model plants and broader physiological analyses further support roles for raffinose and galactinol in membrane stabilisation and protection against oxidative stress [32,33], although these functions have not been systematically validated across commercial edible *Brassica* organs. Consequently, whole-tissue raffinose concentrations may integrate metabolically and spatially distinct pools whose individual physiological roles cannot be inferred from bulk concentration alone [3,8].

Cold exposure currently provides the strongest direct connection between plant physiological state and raffinose abundance in edible *Brassica*. Compound-specific profiling in kale demonstrated increases in raffinose and galactinol during acclimation of intact plants to 2 °C, confirming that raffinose abundance in edible leaves can respond dynamically to growth temperature and physiological state [34]. This observation is consistent with the broader cold-responsive behaviour of the GolS–RFS pathway established in *Arabidopsis* and other plant systems [7,13,22]. Importantly, however, preharvest cold acclimation of an intact plant must be distinguished from refrigeration of harvested vegetables. Direct evidence that raffinose consistently continues to accumulate during commercial cold storage of *Brassica* products remains insufficient. Thus, temperature exposure and acclimation history before harvest should be considered potential determinants of measured RFO composition, whereas the contribution of subsequent cold-chain storage requires dedicated *Brassica*-specific investigation.

Beyond cold, studies across plant species indicate that RFO metabolism responds to drought, salinity, osmotic imbalance, and oxidative stress [2,6,10,22,33]. Direct evidence quantifying these responses in edible *Brassica* vegetables under agronomically realistic conditions is nevertheless scarce. Agricultural practices that alter plant water status, temperature exposure, nutrient availability, or developmental rate may therefore influence RFO profiles, but their relative contributions remain insufficiently resolved. Accordingly, physiological interpretation should follow an explicit evidence hierarchy: (1) Direct measurements in edible *Brassica* tissues provide the strongest basis for food-related conclusions. (2) Studies in *B. napus* provide *Brassica*-specific biological context. (3) Evidence from *Arabidopsis* or other crop species should support plausibility rather than substitute for direct validation in the edible tissue of interest.

## 4. From Plant Stress Metabolite to Food-Quality Trait

Among edible *Brassica* vegetables, kale (*Brassica oleracea* var. acephala) has received comparatively substantial attention with respect to low-molecular-weight carbohydrates (LMWC) and compounds classified as prebiotic carbohydrates. Compositional studies demonstrate genotype-, water-status-, cropping-system-, and germplasm-dependent variation in broader oligosaccharide profiles [4,5,35,36]. However, these studies also reveal a fundamental limitation of the evidence base: raffinose is frequently not quantified as an independent analyte. In Thavarajah et al. [5], the values reported across grouped oligosaccharide fractions spanned 0.8–169 mg 100 g^−1^ fresh weight, but this overall span does not represent individual raffinose concentrations. Specifically, the study reported stachyose + raffinose at 13–169 mg 100 g^−1^, verbascose + kestose at 0.8–39 mg 100 g^−1^, and nystose at ND–10 mg 100 g^−1^. Pathirana et al. [4] similarly reported raffinose together with stachyose, while Thavarajah et al. [37] combined raffinose with fructooligosaccharides, and Bianchi et al. [35] reported total prebiotic oligosaccharides. These studies therefore provide convincing evidence for variability in *Brassica* oligosaccharide composition but not for an equivalently large variation in raffinose itself. The apparent magnitude of variation depends strongly on analyte definition, reporting basis, genotype, and experimental context.

More direct evidence for the dynamic behaviour of raffinose in edible *Brassica* tissues comes from compound-specific profiling. Megías-Pérez et al. [34] used complementary GC-MS and HILIC-MS approaches to characterise LMWC in three commercial kale cultivars during development and cold acclimation. Raffinose and galactinol increased in plants exposed to 2 °C, demonstrating that raffinose abundance in edible leaves responds to physiological state and growth temperature rather than representing a fixed cultivar trait. Earlier work on frost-hardened cabbage likewise identified cold-associated redistribution of soluble carbohydrates, including a physiologically relevant chloroplastic raffinose pool [31]. These *Brassica*-specific observations are consistent with broader evidence for stress-responsive GolS-RFS metabolism across plant species [2,7,10,22], although such studies should support, rather than substitute for, direct evidence from edible tissues.

Compound-specific data reported by Megías-Pérez et al. [34] provide quantitative evidence of raffinose accumulation during preharvest cold acclimation of kale. After 11 days at 2 °C, concentrations in wild cabbage, Frostara and Black Tuscany were 66.9 (60.5–73.3), 50.0 (31.3–68.6) and 48.4 (35.4–61.5) mg g^−1^ dry matter (DM), respectively (means with reported minimum–maximum ranges). Leaf samples collected from two plants per cultivar, treatment and sampling time were pooled before analysis, limiting assessment of independent plant-to-plant variation. At day 11, raffinose in the corresponding 25 °C controls was reported as not detected in wild cabbage and Frostara and below the quantification limit in Black Tuscany; these categories must not be interpreted as measured zero concentrations. A critical distinction is required between preharvest cold acclimation and postharvest refrigeration. Cold exposure of an intact plant can activate acclimation pathways and promote raffinose and galactinol accumulation, as demonstrated directly in kale [34]. By contrast, evidence that commercial refrigeration consistently increases raffinose in harvested *Brassica* vegetables remains insufficient. Hagen et al. [38] found that curly kale stored at 1 °C showed reductions in total soluble sugars and vitamin C, whereas field-retained plants exposed to frost accumulated soluble sugars and dry matter; individual raffinose kinetics were not determined. Thus, preharvest acclimation and postharvest storage cannot be treated as interchangeable metabolic stimuli, and evidence from intact *Brassica* plants or model species [13,31,34] does not establish that raffinose necessarily accumulates during commercial cold storage. Raffinose should therefore currently be regarded as a candidate indicator of physiological cold exposure rather than a validated marker of cold-chain history or freshness.

Agronomic management may also modify raffinose as part of broader metabolic reprogramming. In mizuna (*Brassica rapa* var. nipposinica), GC–MS-based metabolite profiling combined with sensory analysis showed that fertiliser regime altered both metabolic and sensory traits; raffinose, maltose, and malic acid were positively associated with perceived bitterness, whereas allyl isothiocyanate was more directly associated with characteristic pungency [39]. These associations do not demonstrate that raffinose itself is a major bitter tastant. Integrated transcriptomic and metabolomic analysis of green and red mizuna further revealed cultivar-dependent differences in raffinose, sucrose, maltose, trehalose, inositol, glucosinolates, anthocyanins, and phenylpropanoids [40]. Raffinose may therefore co-vary with sensory attributes as part of a broader metabolic phenotype, but its independent contribution to flavour cannot be inferred from correlation alone.

The available evidence likewise does not support simple species-level rankings of raffinose content across broccoli, Brussels sprouts, cabbage, kale, and other *Brassica* vegetables. Comparative α-galactoside profiling confirms genetic variation in raffinose- and stachyose-related composition [41], but much of this evidence derives from seeds or non-equivalent tissues. In edible organs, comparisons are further confounded by developmental stage, water content, source–sink relationships, environmental and postharvest history, and inconsistent reporting as individual raffinose, grouped RFO/FOS fractions, or broader prebiotic-oligosaccharide categories on either fresh- or dry-weight bases [4,5,35,36,37]. The relevant food-quality question is therefore not whether a particular *Brassica* species intrinsically contains “high” or “low” raffinose but how genotype, edible organ, physiological history, postharvest treatment, and analytical methodology jointly determine the RFO profile measured at consumption.

Overall, current evidence supports considering raffinose as a potentially informative food-quality trait but not as a validated standalone marker. Direct studies demonstrate temperature-responsive raffinose accumulation in kale, while broader studies reveal genotype-, management-, and cultivar-associated variation in oligosaccharide and metabolic profiles. Major limitations remain, including frequent analytical grouping of individual RFOs, conflation of preharvest acclimation with postharvest storage, and scarcity of harmonised comparisons across edible *Brassica* organs. The available compositional evidence is therefore summarised in Table 1, which distinguishes individual raffinose measurements from grouped RFO or broader oligosaccharide fractions and highlights differences in biological context, reporting basis, and analytical methodology.

## 5. Nutritional Meaning Beyond a Binary Classification

The nutritional interpretation of raffinose in *Brassica* vegetables requires particular caution because the available evidence derives from markedly different experimental contexts. Gastrointestinal symptoms attributed to RFOs have been documented primarily in studies of legumes, isolated fermentable carbohydrates, or diets specifically designed to manipulate FODMAP exposure [2,23,42]. These findings establish that RFOs can contribute to gastrointestinal symptoms when ingested at sufficient doses by susceptible individuals, but they do not define a *Brassica*-specific dose–response relationship. Direct quantitative assessment is further complicated by the limited availability of compound-specific raffinose data for edible *Brassica* tissues; values reported as combined raffinose–FOS, total RFOs, or broader oligosaccharide fractions cannot be used to calculate raffinose exposure independently. Accordingly, the grouped oligosaccharide ranges discussed in Section 4 cannot be used as individual raffinose exposure estimates [5]. At present, there is therefore insufficient direct evidence to establish either a universal threshold for adverse effects or a typical physiologically relevant dose of raffinose derived specifically from *Brassica* vegetables. A further interpretive problem is the tendency to use raffinose as a proxy for the gastrointestinal relevance of the broader RFO fraction. Raffinose, stachyose, and higher α-galactooligosaccharides share resistance to digestion in the human small intestine, but they are chemically distinct compounds whose concentrations, fermentative behaviour, and contribution to total dietary RFO exposure need not be equivalent [1]. This distinction is particularly important in *Brassica* vegetables, where compound-specific quantitative data remain limited and raffinose and stachyose are frequently reported together. Consequently, gastrointestinal effects attributed broadly to raffinose may in some cases reflect the combined RFO load rather than raffinose exposure itself. The relative quantitative contribution of stachyose, raffinose, and higher RFOs in individual *Brassica* vegetables remains insufficiently resolved and warrants direct compound-specific investigation, particularly when gastrointestinal effects are attributed specifically to raffinose despite incomplete resolution of the total fermentable RFO profile.

The broader physiological rationale for considering raffinose nutritionally relevant is well established. Humans lack sufficient endogenous α-galactosidase activity to hydrolyse the α-galactosidic linkages of raffinose and related RFOs in the upper gastrointestinal tract, allowing these carbohydrates to reach the colon and become available for microbial fermentation [23]. Microbiome studies demonstrate that RFO utilisation is taxon- and enzyme-dependent rather than uniform across the gut microbial community. Recent molecular work in *Bacteroides* species has identified specific determinants of RFO utilisation, providing direct evidence that the capacity to metabolise these substrates varies among bacterial taxa [43]. Multi-donor fermentation studies further indicate substantial inter-individual variation in fermentation outcomes, supporting the view that baseline microbiome composition can modify responses to structurally defined fermentable carbohydrates [44]. These findings establish the biological plausibility of microbiome-dependent raffinose utilisation, but they do not demonstrate that consumption of raffinose within a *Brassica* food matrix produces equivalent effects in vivo.

Related work on non-RFO fermentable polysaccharides further illustrates why microbial outcomes depend on substrate structure and experimental system: Astragalus polysaccharides were examined in diabetic mice [45], whereas blackberry polysaccharides of differing molecular weights were fermented in vitro [46]. These studies broaden the context for carbohydrate–microbiota interactions but do not validate *Brassica*-derived raffinose exposure, RFO-specific analytical methods, or clinical health benefits.

This distinction is particularly important when discussing prebiotic potential. According to the ISAPP consensus definition, a prebiotic must be selectively utilised by host microorganisms and confer a demonstrated health benefit [18]. Evidence that raffinose or related RFOs can be fermented by gut bacteria, alter microbial composition, or contribute to the production of short-chain fatty acids (SCFAs) therefore supports their potential prebiotic relevance but is not, by itself, sufficient to establish a prebiotic health effect. Much of the available evidence derives from in vitro fermentation, animal models, soybean oligosaccharides, stachyose-enriched preparations, or other non-*Brassica* substrates [43,44,47,48]. Such studies provide valuable support, whereas direct intervention studies using analytically characterised *Brassica*-derived raffinose or defined *Brassica* foods remain lacking. A dietary intervention with sulforaphane-rich broccoli sprouts reported changes in bowel habits [49], but the study did not isolate or quantify raffinose as the causal component and cannot support a *Brassica*-RFO-specific effect. Raffinose in *Brassica* should therefore be described as a fermentable carbohydrate with potential prebiotic relevance, rather than as an established prebiotic component of these vegetables.

The same evidence hierarchy is required when interpreting raffinose within the FODMAP framework. Controlled dietary studies and systematic assessments of low-FODMAP interventions provide evidence that restriction of fermentable carbohydrates can alleviate gastrointestinal symptoms in subsets of individuals with irritable bowel syndrome (IBS), although responses vary, the certainty of evidence is heterogeneous, and restriction may adversely affect beneficial microbial populations, including Bifidobacterium, as well as microbial fermentation products [42,50,51]. These clinical findings concern the cumulative fermentable-carbohydrate load of the diet and cannot be translated directly into a universal adverse classification of raffinose-containing *Brassica* vegetables. Conversely, the absence of *Brassica*-specific clinical trials does not demonstrate that these foods are symptom-neutral in all individuals. The most defensible interpretation is therefore context-dependent: gastrointestinal response is likely to reflect the interaction among ingested dose, total meal FODMAP load, individual gastrointestinal sensitivity, microbiome composition and activity, and the physical and chemical characteristics of the food matrix. Microbiome composition may contribute importantly to inter-individual variability, but current evidence does not support treating it as the sole or necessarily primary determinant of raffinose tolerance.

Food processing introduces an additional source of variability between composition at harvest and actual dietary exposure. Because RFOs are water-soluble and susceptible to enzymatic or microbial hydrolysis, soaking, germination, cooking, fermentation, canning, and α-galactosidase treatment can reduce raffinose and related oligosaccharides in a range of food systems [1,2]. However, most quantitative evidence for these effects derives from legumes and seed-based foods rather than edible *Brassica* vegetables. It is therefore inappropriate to transfer specific percentage reductions established in beans or other pulses directly to cabbage, kale, broccoli, or cauliflower. Fermentation and other bioprocessing approaches may plausibly modify RFO exposure in *Brassica*-based foods, but dedicated compound-specific studies are required to determine the magnitude of these changes in individual vegetable matrices. Observed changes must additionally distinguish leaching into discarded cooking water, chemical degradation, enzymatic hydrolysis, and shifts caused by moisture loss; mass balance and the edible portion should therefore be documented before a concentration difference is interpreted as a true RFO loss. Processing is a plausible modifier of *Brassica* RFO exposure, not a uniformly validated reduction method across these products.

Taken together, current nutritional evidence does not support either extreme interpretation of raffinose as an intrinsically anti-nutritional constituent or as a universally beneficial prebiotic. Direct evidence from human consumption of analytically characterised *Brassica*-derived raffinose is currently limited, whereas support from microbiome studies, isolated RFO substrates, other food matrices, and FODMAP interventions is substantially stronger. The nutritional significance of raffinose in *Brassica* should therefore be evaluated through an explicit hierarchy of evidence: direct human studies involving characterised *Brassica* foods or *Brassica*-derived RFO exposure provide the strongest basis for nutritional conclusions; human and microbiome studies using isolated raffinose or related RFOs provide supporting evidence; and in vitro, animal, or non-*Brassica* food studies should be used primarily to establish biological plausibility. Within this framework, dose, food matrix, processing, host sensitivity, and microbiome characteristics collectively determine whether raffinose contributes to beneficial fermentation, gastrointestinal symptoms, or neither at a given dietary exposure.

## 6. Analytical Uncertainty and the Comparability Problem

Reliable interpretation of raffinose concentrations in *Brassica* depends not only on biological variability but also on how the analyte is defined, extracted, quantified, and reported. Published studies do not consistently measure the same chemical fraction: results may refer to individual raffinose, raffinose together with stachyose, combined raffinose–fructooligosaccharide fractions, total RFOs, or broader prebiotic-oligosaccharide pools [4,5,35,37]. Compound-specific profiling in kale further demonstrates that raffinose and galactinol may respond differently from other low-molecular-weight carbohydrates, making grouped reporting particularly problematic for physiological interpretation [34]. Consequently, analyte definition must precede biological comparison because a precise measurement of a combined raffinose–FOS fraction cannot be interpreted as an equally precise measurement of raffinose alone.

Failure to report individual RFOs may have three different causes. Chromatographic co-elution occurs when compounds are insufficiently separated under the selected conditions; incomplete identification occurs when a signal cannot be assigned confidently to a particular compound; and deliberate aggregation occurs when individually quantified analytes are subsequently reported as a combined fraction. A published raffinose–stachyose sum does not, by itself, establish co-elution. These problems require different remedies: optimisation and verification of chromatographic resolution, use of authentic standards and structurally informative confirmation where appropriate, or separate reporting of already resolved analytes [4,5,22,52].

Sample preparation represents a second major source of analytical variability. Recovery of raffinose and related RFOs can be influenced by solvent composition, extraction temperature and duration, solid-to-liquid ratio, homogenisation, and matrix properties [52,53]. Potential metabolic changes between sampling and extraction introduce an additional pre-analytical concern because plant tissues contain α-galactosidases involved in RFO mobilisation and turnover [1,2,23]. Direct quantitative evidence for ex vivo raffinose degradation during extraction of edible *Brassica* tissues remains limited, however, and poorly controlled sample handling should therefore be regarded as a potential rather than universally demonstrated source of bias. Rapid stabilisation and transparent reporting of pre-analytical and extraction conditions remain essential for comparison of fresh, stressed, stored, and processed plant materials [34,52,53,54].

The choice of analytical platform adds a further layer of heterogeneity. Chromatographic and mass-spectrometric approaches differ in sensitivity, selectivity, requirement for derivatisation, susceptibility to matrix effects, structural resolution, calibration behaviour, and accessibility [34,52,54,55]. No platform should therefore be assumed to be universally superior. Direct comparative work on α-galacto-oligosaccharides has demonstrated method-dependent differences in recovery and reproducibility, reinforcing the need for platform-specific validation, authentic standards, recovery assessment, and transparent reporting of analytical performance [52]. The principal analytical approaches applicable to RFO analysis, together with their major strengths, limitations, and recommended roles in *Brassica* research, are summarised in Table 2.

Reporting should specify the individual analytes (or defined sums), authentic standards, calibration, verified separation and recovery, LOD/LOQ, precision, matrix effects, and the FW/DW basis [45,52]. HPLC, high-performance liquid chromatography; RI, refractive index; ELSD, evaporative light-scattering detection; CAD, charged aerosol detection; LC-MS, liquid chromatography–mass spectrometry; MS/MS, tandem mass spectrometry; LOD, limit of detection; LOQ, limit of quantification.

Method performance must be evaluated for the specific analyte and matrix. In dry beans, Pico et al. [52] compared GC–MS and HPAEC–PAD for raffinose, stachyose and verbascose and reported extraction efficiencies of 92.54–107.94%; for the GC–MS procedure, limits of quantification were 4.48–224.31 mg kg^−1^ and intra- and inter-day repeatabilities were below 10%. These figures are study-specific, not universal performance characteristics of either technique or directly transferable to *Brassica*. Ispiryan et al. [58] validated an HPAEC–PAD method for FODMAP quantification in cereals and cereal-based products, illustrating the importance of reporting detection and quantification limits, linearity, repeatability and accuracy. Matrix-specific assessment of analyte recovery, potential interference and calibration remains necessary when applying these approaches to *Brassica* materials.

As summarised in Table 2, no single analytical platform can be regarded as universally optimal for raffinose determination. Method selection should instead reflect the analytical objective, required compound resolution, matrix complexity, and validation requirements. For cross-study comparisons, methodological transparency and compound-specific reporting are therefore at least as important as instrumental sensitivity.

## 7. Breeding and Biotechnological Modification of RFOs in Brassica Crops

This section distinguishes three evidence levels: experiments performed in *Brassica*, breeding proof-of-concept from other species, and proposed *Brassica* applications. In the studies reviewed here, direct interventions in *B. napus* include antisense suppression of galactinol synthesis, seed-specific manipulation of myo-inositol metabolism and *BnaRFS6* overexpression; these are not equivalent to demonstrated CRISPR-based RFO improvement in a *Brassica* cultivar [15,17,24]. The CRISPR RFO experiments discussed below were conducted in soybean, whereas the *Brassica* genome-editing literature supplies platform-level rather than RFO-specific validation [25,26,27,29].

### 7.1. Breeding Objectives for RFOs Modification in Brassica Crops

Nutritional improvement should be defined for a particular product, not equated with constitutive depletion of every RFO: RFOs undergo limited digestion and subsequent microbial fermentation in monogastric consumers and have protective or developmental functions in plants [2,3]. For seed and meal applications, reducing an analytically defined RFO fraction is a plausible compositional objective, but improved feed value requires direct testing: In the soybean RNAi study of Valentine et al. [28], the numerical increase in rooster true metabolisable energy was not statistically significant in the reported comparison (*p* = 0.1802). Thus, lower seed RFO concentration should be treated as an intermediate breeding phenotype, not as proof of nutritional benefit in *Brassica* meal [15,28].

Stress tolerance creates a potentially opposing objective, although the direction and magnitude of the trade-off are context-dependent: BnHSFA4a-mediated regulation of *BnGolS1* has been linked to desiccation responses using rapeseed observations and functional tests in *Arabidopsis*, while *Arabidopsis* seeds lacking RFOs showed delayed germination in darkness [16,61]. These findings justify testing seed vigour and stress performance alongside composition. They do not establish that every reduction in seed RFOs necessarily reduces crop resilience [16,26]. A proposed selection index would therefore combine mature-seed RFO composition with emergence, longevity, reproductive performance and responses to defined water and temperature stresses rather than selecting on sugar concentration alone [2].

Organ identity changes the breeding question: The direct *B. napus* suppression experiment concerns mature seeds, whereas the *BnaRFS6* overexpression study measured stalk carbohydrate composition [15,17]. Neither endpoint can substitute for a validated response in broccoli inflorescences, cabbage heads or another harvested organ. We therefore propose separate targets for low-RFO seed ingredients, stress-responsive vegetative tissues and vegetables consumed after processing. These objectives address different organ-specific physiological requirements [2,15,17]. Table 3 specifies the intended product, target compounds or ratios, physiological constraints and end-use test for each objective. Priorities are recommendations of this review. Nearer-term applications build on direct Brassica compositional evidence; readiness reflects the species, organ and endpoint studied, rather than cultivar availability or a commercial timetable. Establish numerical acceptance criteria for the actual product and environment before selection; no reviewed experiment defines a universal optimum [15,16,17,24,25,26,27,28,61].

### 7.2. Genetic Determinants of RFO Accumulation

Biosynthesis begins when galactinol synthase (GolS) transfers galactose from UDP-galactose to myo-inositol. Raffinose synthase (RFS/RS) then uses galactinol and sucrose to produce raffinose, and stachyose synthase (STS) extends raffinose using another galactinol donor [3]. Consequently, upstream *GolS* suppression and downstream *RFS* or *STS* modification need not produce equivalent changes in individual sugars. Soybean *GolS* editing reduced total RFOs while increasing raffinose, and manipulation of myo-inositol metabolism in *B. napus* redistributed raffinose and stachyose without an apparent reduction in their total [24,26]. Candidate selection must therefore distinguish the desired molecular endpoint—raffinose, stachyose, total RFOs or their ratios—from the name of the edited enzyme [2,26].

Net accumulation also depends on turnover rather than biosynthesis alone: α-galactosidases participate in RFO hydrolysis, and recombinant *Arabidopsis* AtRS4 exhibits both synthetic and hydrolytic activities [22,62]. The latter is biochemical evidence from *Arabidopsis*, not proof that every annotated *Brassica* RFS protein has the same substrate range or reaction balance [17,62]. Increasing degradation in a selected organ is therefore a prospective alternative to biosynthetic suppression but requires enzyme validation, developmental profiling and assessment of reserve mobilisation before a *Brassica* breeding target can be assigned [22,61].

Transport must be separated from local synthesis and turnover. Liu et al. [22] discuss RFO-associated phloem loading in particular plant groups but do not establish an equivalent transport mechanism or a functionally validated RFO-specific transporter target in *Brassica*. It would therefore be premature to nominate a *Brassica* transporter for editing solely from a general polymer-trap model. We propose source–sink sampling and flux measurements before transport is used as a breeding lever. These experiments would test whether an organ phenotype reflects synthesis, import, export or hydrolysis rather than assuming one mechanism from concentration alone [22].

Transcriptional regulation offers a route to conditional rather than constitutive intervention. Lang et al. [16] demonstrated BnHSFA4a interaction with heat-shock elements in the *BnGolS1* promoter and tested desiccation-related function through heterologous expression and complementation in *Arabidopsis*. This supports a specific regulatory connection, not a completely resolved BnHSFA4a–GolS–RFS–STS cascade in every *Brassica* organ. Tissue-restricted or stress-responsive promoter modification is consequently proposed as a way to separate seed composition from vegetative protection, but such separation remains to be demonstrated for RFO breeding in *Brassica* [2,16].

Polyploidy adds a second layer of target selection: *B. napus* has A- and C-subgenomes, and its RFS family contains 13 identified members with six *B. rapa* and six *B. oleracea* entries in Table 1 of [17], despite four *B. rapa* genes in its abstract [17,29]. Family membership does not establish which genes are homeologous counterparts, which have diverged functions, or which supply the RFO pool in the intended organ [17]. We therefore propose identifying syntenic relationships, cultivar-specific target sequences and organ-resolved expression before choosing between homeolog-specific and multiplex interventions. Complete family-wide knockout should not be the default merely because multiple copies exist [17,29]. These are annotation-based family counts, not counts of biochemically validated enzymes. Stress-dependent expression, promoter motifs and phylogenetic grouping prioritise candidates but do not demonstrate non-redundant functions; locus-specific perturbation, substrate testing and complementation are required [16,17,62].

### 7.3. Conventional and Marker-Assisted Breeding

Natural allelic variation provides a demonstrated route to altered seed carbohydrate profiles in soybean, including low-stachyose material identified by germplasm screening and alternative *RS3* alleles informative for breeding. For *Brassica*, we propose beginning with a diverse germplasm panel phenotyped for individual RFOs under comparable developmental and environmental conditions. This would test whether useful natural variation is available in the target organ before a transformation-dependent route is chosen. The soybean studies provide a precedent for this discovery strategy [63,64].

Qiu et al. [64] associated low stachyose with a 33 bp deletion in a stachyose synthase gene and developed a co-dominant indel marker, while Hagely et al. [63] combined informative *RS3* alleles with mutant *RS2* to obtain ultra-low-RFO seed profiles. These studies demonstrate the value of linkage, allele characterisation and marker-assisted crossing in soybean, not transferable *Brassica* marker assays [63,64]. Conventional introgression of a *Brassica* low-RFO allele is therefore a reasonable proposed route only after the allele–phenotype relationship has been established in the target species and breeding material.

QTL mapping and GWAS should similarly be distinguished from functional validation. Lee et al. [65] analysed 220 soybean accessions with 21,317 SNPs across seven environments and identified associations for sucrose and stachyose but excluded raffinose from the GWAS analysis. These soybean studies provide mapping and selection methods but no validated Brassica RFO QTL or marker [63,64,65]. A proposed *Brassica* programme would require replicated phenotyping, control of population structure, independent validation and confirmation that marker effects persist in relevant backgrounds before routine selection.

We propose metabolomics-assisted breeding to complement genetic analysis: Perturbing myo-inositol metabolism in *B. napus* redistributed several carbohydrates, and soybean GolS editing changed raffinose and total RFOs in different directions [24,26]. We propose pairing targeted quantification of galactinol, sucrose and individual RFOs with broader metabolite profiling to detect pathway redistribution and accompanying changes. Multi-environment validation and direct measurements in the harvested product should remain selection requirements rather than be replaced by a metabolomic correlation or a seedling expression signature [2,65].

### 7.4. Genetic Transformation

Direct *Brassica* evidence for down-regulation is provided by Bock et al. [15]: Reintroduction of a galactinol synthase sequence in antisense orientation reduced galactinol and stachyose in mature *B. napus* seeds. This is an antisense transgenic experiment, not a CRISPR knockout and not a demonstration of the hairpin-RNAi strategy used in later soybean work [15,28]. Animal performance and commercial-cultivar stress responses were not established in this experiment. The constitutive CaMV 35S construct used sequence AF106954; mature-seed raffinose did not decrease, and the lowest-stachyose subline retained 36% of the control concentration (a 64% reduction), rather than showing a 36% reduction [15].

Overexpression supports a different objective: *BnaRFS6* overexpression in *B. napus* increased raffinose, glucose and fructose and decreased starch in stalks, whereas the reported subcellular localisation assay used *Arabidopsis* protoplasts [17]. This provides a candidate for investigating increased RFO synthesis but does not demonstrate stress-induced leaf protection or a beneficial seed phenotype [17]. Separately, seed-specific expression of an inositol methyltransferase in *B. napus* decreased phytate by 19–35%, while raffinose decreased and stachyose increased with apparently unchanged total RFOs; the phytate reduction must not be relabelled as an RFO reduction [24].

RNAi proof-of-concept is supplied by soybean *RS2* silencing, which lowered seed raffinose, but the corresponding feeding comparison did not establish a statistically significant energy advantage [28]. Moreover, removal of a selectable-marker locus by segregation does not remove a retained RNAi construct: Marker-free and transgene-free describe different outcomes in this experiment. For *Brassica*, we propose comparing antisense or RNAi suppression with independently generated alleles under matched expression and growth conditions, rather than assuming that all forms of silencing reproduce the same phenotype. The reported feeding-lot comparison gave TME means of 2734 versus 2385 kcal/kg, with *p* = 0.1802; this is a measured nutritional endpoint with an inconclusive treatment comparison, not a demonstrated feeding benefit [28].

The metabolic effects of *BnaRFS6* overexpression and inositol methylation illustrate why a successful transformation is not equivalent to a selectively improved RFO trait [17,24]. Stable transformation also remains coupled to the delivery and regeneration system, whose performance varies with *Brassica* material and experimental protocol [29]. Proposed evaluation should therefore include multiple independent events, segregating controls, compound-resolved composition and the physiological traits relevant to the intended product.

### 7.5. New Genomic Techniques

CRISPR/Cas and multiplex editing provide strong RFO proof-of-concept in soybean, but the metabolic endpoint is target-dependent. Le et al. [26] reported an approximately 35% reduction in total seed RFOs after *GmGOLS1A*/*GmGOLS1B* editing, accompanied by a 41.7% increase in raffinose rather than raffinose depletion. Cao et al. [25] reduced raffinose and stachyose through *RS2*/*RS3* editing, and Lin et al. [27] obtained almost RFO-free soybean seed by combining edits in *RS2*, *RS3*, *RS4* and *STS*. These soybean results identify target combinations for testing in Brassica, where their sugar and agronomic effects require validation [25,26,27].

Site-directed nuclease (SDN) categories SDN-1, SDN-2 and SDN-3 should not be presented as equally demonstrated RFO-breeding routes—the selected soybean studies substantiate nuclease-induced mutagenesis, not a comparative experimental assessment of all three categories [25,26,27]. The *Brassica* platform review distinguishes end-joining-based mutagenesis from donor-dependent repair and insertion approaches, but this technical distinction does not constitute evidence of precise donor-mediated RFO allele replacement or RFO-gene insertion in *Brassica* [29]. Accordingly, loss-of-function testing has the closest cross-crop experimental precedent, whereas targeted sequence replacement and insertion remain proposed RFO applications within the evidence set evaluated here [25,26,29].

Multiplex design is especially relevant to the combination of several functional targets, as demonstrated in soybean, and to the challenge of related gene copies in oilseed rape [27,29]. Nevertheless, simultaneous editing should follow functional prioritisation: The 13-member *BnaRFS* family does not establish that all copies should be disabled or that one conserved guide will produce the desired organ-specific phenotype [17]. A proposed *Brassica* strategy is to compare single-copy, selected homologue-pair and broader combinations while resolving each edited locus and measuring individual RFOs.

Base editing and prime editing extend the design space beyond conventional nuclease mutagenesis in the *Brassica* biotechnology discussion, but the selected RFO experiments do not demonstrate either approach for *Brassica* RFO modification [25,26,27,29]. They are therefore proposed here for testing functionally justified partial-activity or regulatory alleles rather than as proven improvements over knockout. Greater precision in the intended sequence change should not be taken to guarantee fewer metabolic side effects; pathway redistribution and multifunctional enzyme activity remain biological questions regardless of the editing tool [24,26,62].

Cisgenesis and intragenesis remain prospective RFO strategies: the reviewed Brassica studies instead document antisense expression, heterologous metabolic engineering, overexpression and genome-editing platforms [15,17,24,29]. The present evidence therefore does not support ranking cisgenic or intragenic RFO strategies for efficiency, biological safety or regulatory status. A candidate endogenous allele or expression unit would first require functional and organ-specific validation; terminology alone cannot demonstrate the desired nutritional–stress balance.

DNA-free delivery must be distinguished from obtaining transgene-free segregants after a DNA-based editing step: delivery of Cas protein–guide RNA (gRNA) ribonucleoprotein (RNP) complexes and subsequent segregation of an integrated editing construct are different routes discussed for *Brassica* editing. The proposed RFO application is to select the route compatible with the target genotype and available regeneration system and then verify molecularly the final plant. Successful editing in isolated cells is not itself evidence of an inherited, transgene-free *Brassica* RFO trait [29].

### 7.6. Transformation and Regeneration Platforms

*Agrobacterium*-mediated delivery to regenerable explants, including hypocotyl-based systems in oilseed rape, is an established component of *Brassica* biotechnology, whereas its efficiency depends on the genotype and protocol [29]. The *BnaRFS6* study provides an RFO-related example using hypocotyl transformation, but does not establish a universal platform for vegetable *Brassica* genotypes [17]. A matched comparison of cotyledon and hypocotyl explants across target cultivars is therefore needed before ranking their suitability for RFO modification [17,29].

Protoplast-based delivery, including polyethylene glycol (PEG)-assisted introduction of editing reagents and RNP-based approaches, offers an alternative entry point, but whole-plant recovery remains a separate requirement from detecting edits in treated cells. For an RFO project, we propose measuring protoplast survival, cell division, shoot regeneration, rooting, fertility and transmission as separate endpoints rather than reducing platform performance to a single editing percentage. Genotype-dependent regeneration is consequently a selection constraint for the experimental system itself, not merely a final optimisation step after guide design [29]. Reported efficiencies must retain their original denominators: The 50.5% and 46.7% mean target-editing values in Cao et al. [25] came from a soybean hairy-root comparison of two construct architectures, not from *Brassica* transformation or fertile-plant recovery. Similarly, an explant transformation rate, an allele-editing frequency and a regeneration rate answer different operational questions and should not be pooled into one performance estimate [25,29]. The proposed reporting standard is the number of independently recovered, fertile, molecularly characterised lines per treated explant or protoplast input, accompanied by intermediate-stage counts and the genotype identity.

### 7.7. Candidate Targets and Proposed Strategies

Table 4 compares the experimental intervention studies, Table 5 links proposed strategies to molecular candidates and validation requirements, and Table 3 prioritises objectives by intended product. Table 3 and Table 5 define research priorities. Direct *Brassica* evidence comprises antisense galactinol suppression, altered inositol metabolism and *BnaRFS6* overexpression, while most precise-editing precedents are from soybean [15,17,24,25,26,27].

RFS/RS, raffinose synthase; STS, stachyose synthase; GolS, galactinol synthase; RNP, ribonucleoprotein. gRNA, guide RNA; SDN, site-directed nuclease. All proposed *Brassica* RFO strategies require validation; platform feasibility and cross-species precedent are not equivalent to a validated breeding outcome. Published sequence accessions identify clones or constructs and must not be treated as current genome loci without sequence alignment. ZS locus identifiers are retained as reported in [17]; that study does not specify an exact assembly release. Same-clade A- and C-subgenome loci are candidate relationships, not proven functional homeolog pairs. No functionally validated *Brassica* STS locus is assigned from this evidence set [15,16,17,62].

The proposed order of work is to define the product-level phenotype, establish compound-specific baseline variation, validate candidate gene function in the relevant organ, test the smallest justified intervention and then assess seed performance, stress responses and agronomic stability. This sequence is intended to prevent a change in one sugar or a high cell-editing frequency from being mistaken for a nutritionally improved and physiologically robust *Brassica* crop; the need for these separate endpoints is illustrated by contrasting sugar responses, context-dependent germination effects and the limitations of feeding evidence in the selected studies [24,25,26,28,61]. Product-specific acceptance thresholds should be defined before selection: the reviewed evidence does not establish a universal optimum or safe minimum RFO concentration. For seed ingredients, GolS suppression is the direct *Brassica* experimental anchor; leaf stress protection and consumer-health applications require additional candidate discovery and end-use testing [15,16,28,34,61].

## 8. A Three-Tier Interpretive Framework

**Figure 2:**
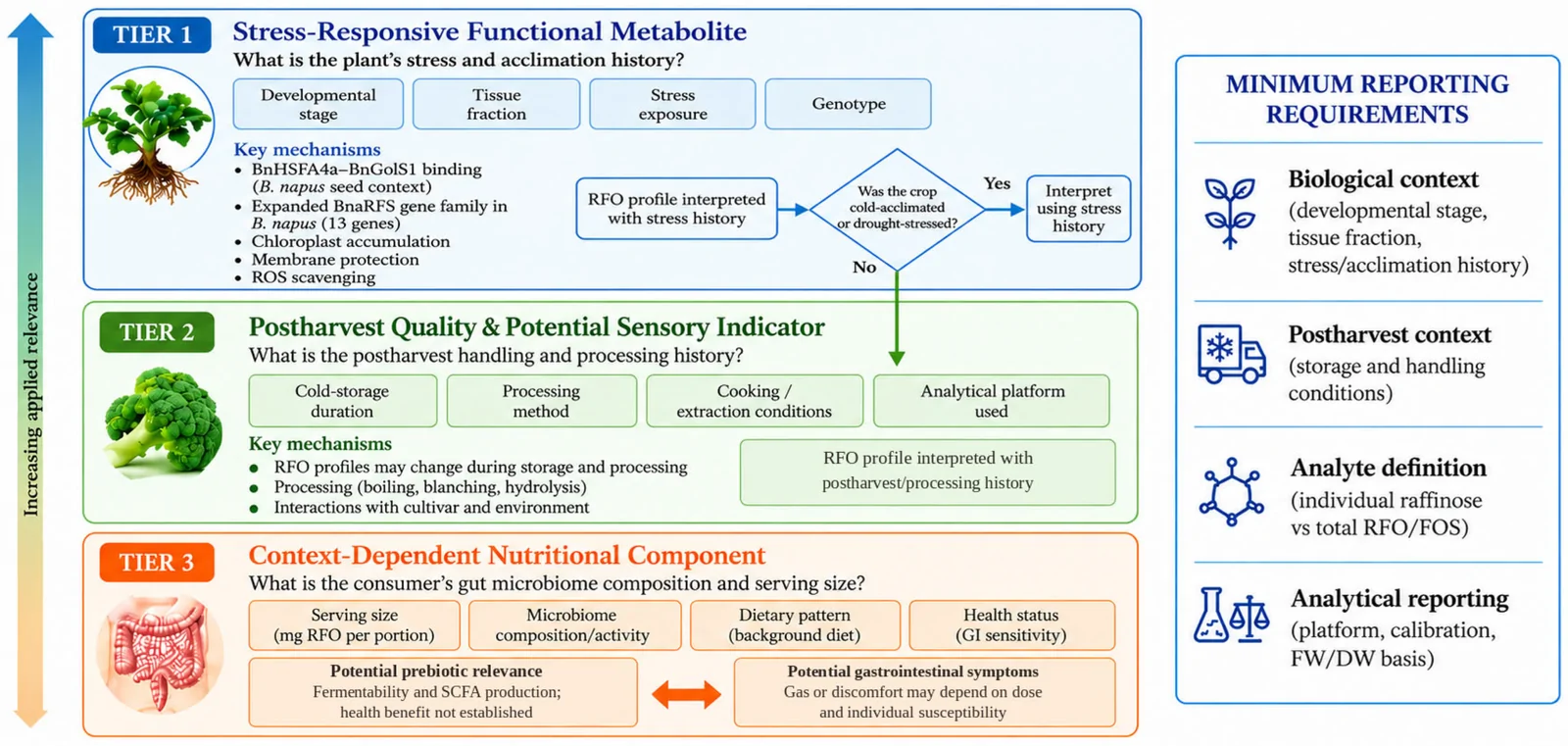
Proposed three-tier framework for interpreting raffinose in *Brassica*. The tiers organise qualitative, context-aware interpretation rather than numerical adjustment of measured RFO concentrations. The terms used for the stress and postharvest profiles do not indicate a defined correction formula, and neither a validated freshness marker, a diagnostic test nor a health claim is established. Physiological, food-quality and nutritional evidence must retain their respective experimental contexts [2,18,34].

The evidence reviewed above indicates that raffinose in *Brassica* cannot be interpreted within a single biological or nutritional context. Its significance depends on the physiological state of the plant, the postharvest and processing history of the edible tissue, and the conditions under which dietary exposure occurs. To integrate these dimensions while maintaining a clear distinction between direct and supporting evidence, we propose a three-tier interpretive framework encompassing plant stress physiology, postharvest and food-quality processes, and context-dependent nutritional effects (Figure 2).

The first tier treats raffinose as a dynamic, stress-responsive metabolite, supported by direct kale acclimation studies and by molecular evidence with distinct species and tissue scopes [16,17,34]. We propose reporting growth temperature, organ, developmental stage and stress history as minimum metadata. Identification of 13 *BnaRFS* genes is a genomic result, not proof that all 13 contribute equally to raffinose accumulation in each organ [17].

The second tier treats raffinose as a candidate food-quality-related indicator rather than a validated standalone marker of freshness, cold-chain integrity, sweetness or fermentation performance. Direct kale cold-acclimation evidence and reported storage or sensory associations do not establish those applications [34,38,39,40]. We therefore propose separating preharvest exposure from postharvest treatments and testing predictive performance directly before a quality-marker claim is made.

The third tier addresses nutritional interpretation in relation to dose, food matrix and host response; evidence of microbial substrate utilisation does not alone establish a prebiotic health benefit [18,20]. A typical *Brassica* raffinose dose cannot be derived from combined raffinose–stachyose or raffinose–FOS values, and adverse or beneficial effects should not be inferred from those grouped measurements [2,5,37]. The proposed framework therefore calls for simultaneous attention to plant history, product handling and measured exposure while allowing each tier to be investigated independently with appropriately bounded conclusions.

Two contrasting examples illustrate the practical interpretation of the three tiers in Figure 2. First, in kale acclimated as intact plants to 2 °C, compound-specific increases in leaf raffinose and galactinol provide direct evidence of stress-responsive metabolism (Tier 1) [34]. These findings should not be interpreted as evidence that postharvest refrigeration increases raffinose or that raffinose predicts sensory quality (Tier 2); nor do they establish a prebiotic health benefit or gastrointestinal response after consumption (Tier 3). Second, consider rapeseed meal hypothetically produced from *B. napus* seeds with reduced RFOs through suppression of galactinol synthesis. The reported antisense experiment demonstrates reduced galactinol and stachyose in mature seeds [15] but does not establish the composition or nutritional performance of the processed meal. Application of Tier 1 would therefore require evaluation of seed physiology and stress-related traits alongside individual RFOs; Tier 2 would require compound-specific analysis of the final meal and documentation of processing conditions; and Tier 3 would require product-relevant feeding studies before improved nutritional value could be claimed. In both examples, the tiers organise complementary contextual evaluations rather than provide numerical corrections to measured RFO concentrations.

## 9. Critical Evidence Limitations and Future Research Priorities

A central limitation of the current raffinose literature is not simply the scarcity of data but the unequal evidential weight of the available studies. Direct evidence from edible *Brassica* tissues and products remains comparatively limited and should be distinguished from evidence generated in *B. napus* seeds or other *Brassica* tissues, as well as from supporting studies conducted in *Arabidopsis*, soybean, maize, legumes, or purified raffinose systems. The same distinction is essential in nutritional interpretation: Evidence that isolated raffinose can be utilised by gut microorganisms does not constitute direct evidence of a prebiotic health effect following consumption of a *Brassica* vegetable. Throughout the field, these evidence levels have frequently been treated as interchangeable, contributing to over-extrapolation across species, tissues, experimental conditions, and nutritional contexts [2,10,22,23,31]. Future research should therefore prioritise biologically proximal evidence: direct measurements in edible *Brassica* organs and foods, followed by *Brassica*-specific studies, with evidence from model species used explicitly as support rather than as a substitute for crop-specific validation.

One of the most immediate barriers is the lack of analytical harmonisation. Published studies differ in extraction procedures, enzyme-inactivation protocols, analytical platforms, calibration strategies, reporting basis, and even the definition of the analyte itself. Critically, some studies quantify individual raffinose, whereas others report combined raffinose–FOS, raffinose–stachyose, total RFOs, or broader prebiotic-oligosaccharide fractions. Future studies should therefore quantify individual RFOs rather than treating raffinose as a surrogate for the broader α-galactooligosaccharide fraction, particularly when dietary exposure, gastrointestinal tolerance, or prebiotic relevance is being evaluated. As shown in Section 4 and Table 1, values derived from grouped oligosaccharide fractions cannot be read as individual raffinose concentrations [5]. Related kale studies likewise demonstrate substantial genotype- and management-dependent variability in broader carbohydrate profiles but often do not resolve individual raffinose concentrations [4,5,35,36,37]. Consequently, the first practical priority should be the development of harmonised, compound-specific analytical workflows for edible *Brassica* matrices, including explicit analyte definition, validated extraction and α-galactosidase-inactivation procedures, inter-laboratory cross-validation, certified reference materials, and mandatory reporting on clearly defined FW or DW bases. Without this foundation, increasing the volume of published data may add numerical precision without improving biological comparability.

A second priority is to resolve the relative contributions of genotype, environment, developmental stage, and stress history to RFO variability. Direct studies in kale provide evidence that raffinose and galactinol respond to cold acclimation, while broader oligosaccharide profiles vary with genotype, water availability, and cultivation system [4,5,34,37]. However, the available datasets are insufficient to establish a reliable quantitative range for individual raffinose across *Brassica* germplasm. Future studies should therefore combine compound-specific RFO profiling with multi-location and multi-season experimental designs, concurrent physiological phenotyping, and detailed environmental metadata. Integrating these measurements with genome-wide genotyping or targeted GolS/*RFS* resequencing could ultimately enable genotype-by-environment-aware association studies in vegetable *Brassica*. Multi-environment soybean association analysis supplies a methodological precedent but not direct evidence for *Brassica* RFO genetics [65]. The key objective is therefore not to explain an assumed extreme range in raffinose concentration but to determine how much of the genuine variation in individually quantified raffinose and other RFOs can be attributed to genotype versus physiological and environmental context.

Postharvest behaviour constitutes a third major limitation of the current evidence base. Direct *Brassica* evidence supports raffinose accumulation during cold acclimation of intact plants, but this should not be conflated with commercial postharvest refrigeration [31,34]. Studies in model plants, including the cold-acclimation work of Klotke et al. [13] in *Arabidopsis thaliana*, provide important support for stress-responsive raffinose metabolism but do not demonstrate postharvest accumulation in harvested *Brassica* vegetables. Indeed, available kale storage data indicate that cold storage and field frost exposure can produce different carbohydrate responses, and individual raffinose kinetics under realistic supply-chain conditions remain poorly characterised [38]. Future work should therefore quantify individual RFOs in commercially relevant *Brassica* products using time-resolved designs that separate preharvest acclimation from postharvest refrigeration and systematically vary temperature, storage duration, controlled or modified atmosphere, and processing history. Until such data are available, raffinose should be regarded as a candidate marker of physiological cold exposure rather than a validated marker of cold-chain integrity.

The limitations of the nutritional evidence base are equally important and require particularly strict application of the evidence hierarchy. Microbiome research demonstrates that members of the human gut microbiota possess enzymatic systems capable of utilising RFOs, and recent work has clarified specific α-galactosidase-dependent pathways involved in RFO utilisation by *Bacteroides* species [43]. Such findings strengthen the biological plausibility of raffinose as a fermentable microbiota substrate, while broader microbiome and prebiotic research indicates substantial inter-individual variation in carbohydrate utilisation [18,20]. However, these data do not demonstrate that raffinose consumed within a *Brassica* food matrix confers a prebiotic health benefit, nor do they establish *Brassica*-specific dose thresholds for gastrointestinal intolerance. The studies evaluated here do not establish a *Brassica*-food-specific raffinose dose–response relationship; this finding does not imply that evidence from other fermentable-carbohydrate interventions is irrelevant to estimating overall dietary exposure or potential gastrointestinal symptoms. A high-priority clinical programme should therefore test analytically defined *Brassica* preparations in both healthy adults and individuals with IBS, with realistic dietary exposures and endpoints encompassing gastrointestinal symptoms, microbial composition and function, and SCFA production.

The final priority is to translate molecular findings into product-specific *Brassica* breeding without conflating experimental systems. Antisense manipulation and *BnaRFS6* overexpression establish direct *Brassica* pathway responsiveness, whereas selected soybean CRISPR studies establish cross-species proof-of-concept for targeted RFO modification [15,17,25,26,27]. We propose integrating organ-resolved expression, compound-specific metabolomics, validated allele combinations and stress phenotyping before deployment. Delivery, regeneration and recovery of fertile plants should be evaluated for the actual target genotype rather than inferred from cell-level editing or a permissive reference cultivar [29]. The resulting objective is context-specific RFO optimisation, with nutritional performance and crop resilience tested as separate outcomes. Table 6 summarises the remaining priorities.

Taken together, these priorities define a sequential research strategy. Analytical harmonisation must precede large-scale compositional comparison; compound-specific multi-environment phenotyping must precede robust genetic association. Direct postharvest studies must precede the use of raffinose as a supply-chain quality marker. Brassica-specific dietary interventions are needed to attribute health effects to particular Brassica foods, while existing evidence on fermentable carbohydrates can still inform dietary-exposure and symptom-risk hypotheses. Similarly, findings from *B. napus* and model species should guide hypothesis generation but should not substitute for validation in edible *Brassica* tissues and products. This evidence-weighted approach would move the field beyond both the traditional anti-nutritional paradigm and its equally premature opposite—the assumption that raffinose is universally beneficial—towards a context-dependent interpretation grounded in species, tissue, analytical definition, processing history, dose, and consumer response.

## 10. Conclusions

Raffinose in *Brassica* should be interpreted as a context-dependent component of RFO metabolism rather than assigned a universal nutritional label. Its significance depends on organ identity, physiological and postharvest history, analytical definition, processing, dietary exposure, and host response. Raffinose may reflect plant stress physiology and metabolic adjustment, but its concentration alone does not establish food quality, prebiotic benefit, gastrointestinal relevance, or anti-nutritional effect. A major limitation of the current evidence base is the frequent use of grouped oligosaccharide fractions, which prevents reliable attribution of biological or nutritional effects to individual compounds. Compound-specific analysis and explicit reporting of individual RFOs are therefore essential for meaningful comparison across *Brassica* tissues, products, and experimental conditions.

This context dependence is equally important for breeding and biotechnology. Modification of the RFO pathway is feasible, but reducing total RFOs does not necessarily reduce raffinose, and compositional change alone does not demonstrate nutritional or agronomic improvement. Future *Brassica* improvement should therefore be based on a clearly defined product- and organ-specific objective, followed by functional validation of molecular targets, compound-resolved phenotyping, and direct assessment of physiological performance and end-use quality. In polyploid crops, target selection must additionally account for gene-family complexity and genotype-dependent regeneration.

The central conclusion is that RFO optimisation in *Brassica* should not aim at universal depletion but at controlled adjustment of composition for a defined organ, physiological context, and use. The key question is therefore not simply how much raffinose is present but which RFO is present, where, under what conditions, and with what biological consequence.

## Figures and Tables

**Table 1:** Compositional evidence distinguishing individual raffinose from grouped fractions.

Study and Material	Reported Analyte and Result	Context and Reporting Basis	Interpretive Limit
Thavarajah et al. [36] 25 kale genotypes	Broad prebiotic carbohydrates: approximately 0.4–6.7 g 100 g^−1^	Field-grown edible leaves; fresh weight (FW)	Not an individual raffinose concentration range
Pathirana et al. [4] 10 kale genotypes	Raffinose + stachyose: 9–23 mg 100 g^−1^ in controls; 9–32 under moisture stress	80% versus 40% soil moisture for two weeks; values reported per 100 g FW on an 85% moisture basis	Raffinose and stachyose are grouped; cannot infer the response of either separately
Thavarajah et al. [5] 6 cultivars × 6 cover-crop systems	Raffinose + stachyose: 13–169 (mean 73) mg 100 g^−1^ FW; verbascose + kestose: 0.8–39; nystose: ND–10 mg 100 g^−1^ FW	108 observations (6 cultivars × 6 systems × 3 replicates); harvested at physiological maturity (~7 weeks after transplanting); HPAEC-PAD; FW (reported 85% moisture)	Combined raffinose + stachyose is not individual raffinose; mean 73 mg 100 g^−1^ refers to the combined fraction.
Megías-Pérez et al. [34] 3 commercial kale cultivars	Individual raffinose at day 11 (2 °C), mg g^−1^ DM: wild cabbage 66.9 (60.5–73.3); Frostara 50.0 (31.3–68.6); Black Tuscany 48.4 (35.4–61.5). Mean (min–max).	Intact plants: 59 days at 25 °C, then 0, 3, 7 or 11 days at 2 °C vs. 25 °C; leaf samples from two plants per cultivar/treatment/time pooled; HILIC-ESI-TOF MS (quantification), GC-MS (identification).	Compound-specific results from Table 4 of the original study; pooling limits the interpretation of independent biological variability; preharvest acclimation, not postharvest storage.
Thavarajah et al. [37] 18 cultivars and 35 accessions	Combined raffinose/FOS: 0–11.2 mg 100 g^−1^ in commercial cultivars	Organic cultivation; results reported per 100 g FW	Combined category, not individual raffinose
Bianchi et al. [35] 7 kale genotypes	Total prebiotic oligosaccharides: 4.2–10.8 g 100 g^−1^ dry weight (DW)	Germplasm selected for organic cultivation	Not raffinose-specific; not directly comparable with FW values
Santarius and Milde [31] Cabbage leaves	Raffinose redistribution with hardening/dehardening	Subcellular fractions, including chloroplasts	Physiological localisation, not a whole-food concentration dataset

FW, fresh weight; DW/DM, dry weight/dry matter; FOS, fructooligosaccharides; ND, not detected; HPAEC-PAD, high-performance anion-exchange chromatography with pulsed amperometric detection. Grouped fractions must not be treated as individual raffinose. For Megías-Pérez et al., the reported concentrations are means with minimum–maximum ranges from Table 4 of the original study; leaf samples were pooled before analysis.

**Table 2:** Analytical approaches for RFO determination and their implications for comparison.

Approach	Strength and Appropriate Use	Principal Limitation	Selected References
**HPAEC-PAD**	Targeted separation and quantification of underivatised carbohydrates; individual RFOs where chromatographic resolution is verified.	Co-elution, matrix interferences and non-structural detection; verify assignments with authentic standards, assess recovery, LOD/LOQ and precision.	[52,56,57,58]
**GC-MS**	Individual RFO profiling supported by derivatisation and mass-spectral information; suitable for validated targeted analysis.	Extraction and derivatisation can alter recovery and reproducibility; isomer assignment, matrix effects, LOD/LOQ and precision require validation.	[34,52]
**HPLC-RI/ELSD/CAD**	Routine carbohydrate quantification when individual analytes are chromatographically resolved and compound-specific standards are available.	Detector signals do not establish structural identity; co-elution, detector-dependent response, matrix effects and calibration require assessment.	[56,57,59]
**HILIC-MS and LC-MS(/MS)**	Targeted carbohydrate profiling with sensitive detection; MS/MS and authentic standards can strengthen structural assignments.	Retention time or accurate mass alone may not distinguish isomers; ion suppression, standards, diagnostic fragments and calibration must be assessed.	[34,60]
**Enzymatic** **assays**	Rapid screening and complementary measurement of suitable carbohydrate fractions.	Enzyme selectivity and completeness of hydrolysis must be established; individual RFOs may not be resolved.	[1,56]

**Table 3:** Product-specific breeding objectives and practical priorities.

Priority and Intended Product	Compositional Target	Physiological Constraints	Required End-Use Outcome and Readiness
**1. *B. napus* seed meal or protein ingredient**	Lower sum of individually measured seed RFOs, especially stachyose; monitor raffinose separately and the sucrose/RFO ratio	Retain desiccation tolerance, emergence, longevity, oil/protein yield and vegetative stress performance	Demonstrate improved digestibility or energy utilisation in the intended product. Nearer-term compositional follow-up to *Brassica* seed suppression [15]; processed-meal benefit is not established [24,28].
**2. Vegetable rapeseed flowering stalk**	A defined glucose, fructose, sucrose and raffinose profile; no assumption that more raffinose means sweeter taste	Retain marketable yield, texture and appropriate starch/carbon allocation	Demonstrate sensory and processing improvement. Direct stalk-composition evidence exists [17], but palatability remains an end-use test rather than an inference from sugars [17,39].
**3. Stress-resilient leafy *Brassica***	Stress-induced leaf raffinose and galactinol relative to baseline, while retaining the intended seed composition	Measure stress survival/recovery and marketable yield without growth or seed penalties	Longer-term proposal: discover and validate leaf candidates and test performance under defined stresses. Seed regulation and cold-associated accumulation do not demonstrate a protective leaf intervention [16,34,61].
**4. Food for a defined consumer or processed ingredient**	For tolerance: lower RFO dose per prepared serving. For fermentability: define retained compound-specific dose rather than maximise total RFOs	Retain agronomic, sensory and nutritional quality; account for cooking and fermentation	Longer-term: demonstrate gastrointestinal tolerance or an appropriate host health outcome in the specified food. No universal low-RFO threshold or *Brassica* prebiotic target is established [18,28,34,50].

**Table 4:** Comparative experimental interventions affecting RFO metabolism.

Study and Evidence Tier	Species and Genotype	Target and Intervention	Organ Analysed	Individual Sugar Response	Total RFO Response	Measured Outcome and Interpretation
**Bock et al.** [15] ***Brassica***	*B. napus* Westar; selected lines 1 and 28 and sublines	*GolS* AF106954; antisense transgene under CaMV35S promoter	Developing and mature seeds	Mature seeds: stachyose down to 36% of WT in the lowest subline; galactinol down to ~50% of WT there; raffinose not reduced; sucrose slightly higher	Reduced-RFO lines reported; no single total-RFO percentage pooled across events	Developmental and line dependence; no feeding test or demonstrated benefit in processed meal; constitutive suppression is not seed-specific
**Zhou et al.** [17] ***Brassica***	*B. napus* Gaoyousuan No. 1; OE-1/OE-2	*BnaRFS6* (BnaC02G0100500ZS); stable overexpression	Bolting stalks	Raffinose, glucose and fructose increased; starch decreased; sucrose assayed	Individual stachyose and an all-RFO total not reported	Stalk carbohydrate phenotype; no demonstrated seed benefit, leaf stress protection or sensory improvement. Localisation tested in *Arabidopsis* protoplasts
**Dong et al.** [24] ***Brassica***	*B. napus* Westar; napin-IMT and phaseolin-IMT lines	Heterologous myo-inositol methyltransferase; seed-specific napin/phaseolin promoters	Developing and mature seeds; sugar profiles in phaseolin-IMT lines	Raffinose and galactinol decreased; stachyose, sucrose and galactose increased	Overall RFO balance apparently unchanged; raffinose/stachyose ratio altered	Phytate decreased 19–35%; inorganic phosphate increased 10–31%; fresh-seed germination unaffected. Not direct RFO-enzyme editing or a feeding test
**Cao et al.** [25] **Soybean proof-of-concept**	*G. max* Williams 82; *rs2rs3* double mutant	*RS2* (Glyma.06G179200), *RS3* (Glyma.05G003900); multiplex CRISPR/Cas9	Mature seeds	WT → double mutant, % seed DW: raffinose 1.0 → 0.2; stachyose 4.6 → 2.9; sucrose 7.7 → 10.2	Calculated raffinose + stachyose: 5.6 → 3.1% DW (≈45% lower); not a directly measured all-RFO total	No detected greenhouse growth, seed-weight or per-plant yield penalty; no feeding test. Hairy-root editing rates are not fertile-plant recovery rates
**Le et al.** [26] **Soybean proof-of-concept**	*G. max* DT26 for seed profiles; Maverick also transformed	*GmGOLS1A* (Glyma.03G222000), *GmGOLS1B* (Glyma.19G219100); dual-target CRISPR/Cas9	Mature seeds	Double mutants: raffinose +41.7%; stachyose −35.4%; verbascose slightly reduced; sucrose change not significant	64.7 → 41.95 mg/g DW (−35.2%)	No detected germination or morphology difference in tested conditions; protein/fat increases concerned selected single mutants. End-use nutritional benefit not tested
**Lin et al.** [27] **Soybean proof-of-concept**	*G. max*; cultivar NV; double and quadruple mutants	*RS2*, *RS3*, *RS4*, STS; pooled multiplex CRISPR/Cas9	Seeds of characterised progeny	*rs2*/*rs3*: raffinose and stachyose reduced; *rs4*/sts: stachyose reduced; exact individual values NV	Almost eliminated in rfo-4m; exact percentage NV	Seed metabolome shifted, including other oligosaccharide-related metabolites. Physiological and nutritional endpoints NV in the accessible publisher abstract
**Valentine et al.** [28] **Soybean proof-of-concept**	*G. max* Maverick; *RS2* RNAi line	*RS2* (older annotation Glyma06g18890); RNAi silencing	Mature seeds; full-fat soybean meal in rooster assay	Feeding lot, % seed weight: raffinose 0.63 → 0.11; stachyose 3.79 → 1.21; galactinol 0.03 → 0.31; sucrose 6.95 → 9.6	Calculated raffinose + stachyose: 4.42 → 1.32% (≈70% lower); not a measured all-RFO total	TME 2385 → 2734 kcal/kg (paper Table 4), *p* = 0.1802, *n* = 7: higher mean, no significant advantage. RNAi is not CRISPR; marker-free is not transgene-free

Directions refer to the study-specific control. WT, wild type; DW, dry weight; TME, true metabolizable energy; NV, not verified in the accessible primary source. Arrows (→) indicate changes from the control or wild-type values to those of the modified genotype. Calculated sums comprise raffinose plus stachyose only. Galactinol is not an RFO. Values from different organs, developmental stages, seed lots and reporting bases are not pooled. In [26], DT26 provided the reported seed-composition comparison; transformation of Maverick does not make the numerical result a pooled cultivar estimate. For [27], the accessible publisher abstract establishes the qualitative intervention response but does not verify cultivar identity, exact sugar values or physiological endpoints.

**Table 5:** Candidate RFO targets and proposed strategies for *Brassica* breeding.

Breeding Objective	Proposed Intervention	Evidence Basis and Status	Required Validation
**Lower** **seed RFOs**	Edit seed-expressed *RFS*/*STS* candidates or their validated homologs Direct seed anchor: *GolS* antisense sequence AF106954 [15]. Map the construct to cultivar loci before targeted editing. *AtRS4*-related exploratory loci: BnaA09G0022400ZS and BnaC09G0006500ZS [17,62]; neither is validated here as *Brassica* STS.	Prospective in *Brassica*. RS multiplex editing is demonstrated in soybean [25,27]; *Brassica* antisense proof targets *GolS* [15] AF106954 has direct seed-response evidence but no verified current-genome crosswalk. The two ZS loci group with *AtRS4* (AT4G01970); seed activity and functional homeology remain unverified [15,17,62].	Individual sugars, total RFOs, sucrose, seed vigour and product-level nutritional endpoints
**Preserve** **stress** **tolerance**	Modify promoters or restrict suppression to the intended seed stage or tissue Regulatory pair: *BnGolS1* (FJ407183) and *BnHSFA4a* (HQ435241), retained as cloned-sequence accessions rather than inferred genome loci [16].	Regulatory and physiological rationale, not demonstrated uncoupling in *Brassica* [16,61] Seed expression and promoter binding support a specific connection; endogenous locus mapping and *Brassica*-specific causal uncoupling are unresolved [16].	Vegetative stress responses, desiccation, emergence, longevity and yield across conditions
**Increase** **stress-** **responsive** **leaf raffinose**	Activate validated *GolS*/*RFS* targets conditionally rather than assuming constitutive overexpression is optimal Strongest vegetative compositional anchor: *BnaRFS6*, BnaC02G0100500ZS [17]; the demonstrated phenotype concerns stalks, so leaf use remains proposed.	Prospective for leaves. *BnaRFS6* overexpression changes stalk sugars; *BnHSFA4a* evidence is partly heterologous [16,17] ZS annotation namespace, assembly release unspecified in [17]. *AtRS6*-related (AT5G20250); BnaA02G0084600ZS is a related A-subgenome candidate, not a validated equivalent. Expression evidence includes seedling/bolting leaves, stems and buds in lines No. 51/106 [17].	Leaf-specific expression, carbohydrate partitioning and direct stress-performance benefit
**Obtain a final line without an editing transgene**	Use Cas–gRNA RNP delivery or segregate an integrated editing construct	*Brassica* platform options, not RFO-specific proof. Only the former route is DNA-free delivery [29]	Fertile-plant recovery, inheritance and molecular checks for the intended final genotype
**Modify** **a polyploid** **target set**	Use multiplex guides for functionally justified homologue combinations	Prospective for *Brassica* RFOs; supported by soybean multiplex results and *Brassica* platform experience [27,29]	Resolve each locus, allele dosage and retained activity Distinguish syntenic homeology from same-clade homology; compare locus-resolved single and combined perturbations before claiming non-redundant function [17,29,62].
**Limit** **unwanted** **metabolic** **effects**	Test base editing, prime editing or regulatory changes for a justified partial-function allele	Prospective; no guarantee of reduced pleiotropy. Metabolic redistribution occurs after pathway perturbation [24,26]	Compare independent alleles by targeted and broader metabolomics plus physiological testing

**Table 6:** Evidence limitations and priorities for further investigation.

Evidence Domain	Limitation of the Reviewed Material	Proposed Priority	Selected Basis
**Nutritional** **effects**	Fermentation evidence does not establish *Brassica* food-specific clinical benefit or adverse-dose threshold	Characterised food interventions with realistic servings and measured host outcomes	[2,18,20]
**Spatial** **physiology**	Whole-tissue concentrations do not resolve the function of individual pools	Subcellular fractionation and spatially resolved profiling	[22,31]
**Analytical** **comparability**	Individual and grouped oligosaccharide fractions are not interchangeable	Validate extraction, resolution and recovery; report individual sugars and FW/DW basis	[5,34,37]
**Genetic and** **environmental** **effects**	Compositional variation alone does not establish a validated selection marker	Replicated multi-environment phenotyping followed by independently validated genetic associations	[63,64,65]
**Postharvest** **behaviour**	Cold acclimation of intact plants does not establish storage kinetics	Time-resolved measurements across actual supply-chain conditions	[34,38]
**Breeding and** **regeneration**	Cross-species editing success and cell-editing rates do not establish *Brassica* cultivar performance	Recover fertile lines and jointly test composition, stress resilience and product quality	[17,25,29]

Note: Priorities are proposed by this review. The evidence gaps refer to the studies included in this review, not to an exhaustive current-literature search.

## Data Availability

No new data were created or analyzed in this study. Data sharing is not applicable to this article, as this is a review based on the previously published literature.
